# Functions of p53 in pluripotent stem cells

**DOI:** 10.1007/s13238-019-00665-x

**Published:** 2019-11-06

**Authors:** Xuemei Fu, Shouhai Wu, Bo Li, Yang Xu, Jingfeng Liu

**Affiliations:** 1 The Eighth Affiliated Hospital, Sun Yat-sen University, Shenzhen, 518033 China; 2 Center for Regenerative and Translational Medicine, Guangdong Provincial Academy of Chinese Medical Sciences, The Second Affiliated Hospital of Guangzhou University of Chinese Medicine, Guangzhou, 510632 China; 3 Department of Biochemistry and Molecular Biology, Zhongshan School of Medicine, Sun Yat-sen University, Guangzhou, 510080 China; 4 Cancer Research Institute, Guangdong Provincial Key Laboratory of Tumor Immunotherapy, School of Basic Medical Sciences, Southern Medical University, Guangzhou, 510515 China

**Keywords:** p53, embryonic stem cells, induced pluripotent stem cells, genetic stability, metabolism

## Abstract

Pluripotent stem cells (PSCs) are capable of unlimited self-renewal in culture and differentiation into all functional cell types in the body, and thus hold great promise for regenerative medicine. To achieve their clinical potential, it is critical for PSCs to maintain genomic stability during the extended proliferation. The critical tumor suppressor p53 is required to maintain genomic stability of mammalian cells. In response to DNA damage or oncogenic stress, p53 plays multiple roles in maintaining genomic stability of somatic cells by inducing cell cycle arrest, apoptosis, and senescence to prevent the passage of genetic mutations to the daughter cells. p53 is also required to maintain the genomic stability of PSCs. However, in response to the genotoxic stresses, a primary role of p53 in PSCs is to induce the differentiation of PSCs and inhibit pluripotency, providing mechanisms to maintain the genomic stability of the self-renewing PSCs. In addition, the roles of p53 in cellular metabolism might also contribute to genomic stability of PSCs by limiting oxidative stress. In summary, the elucidation of the roles of p53 in PSCs will be a prerequisite for developing safe PSC-based cell therapy.

## The Promise and Challenge of Pluripotent Stem Cells in Human Cell Therapy

Human pluripotent stem cells (PSCs) can undergo unlimited self-renewal and differentiate into all cell types of the human body. Therefore, as a renewable source of various functional cells, PSCs hold great promise for the cell therapy of major human diseases such as neural degenerative diseases, macular degeneration, heart failure and type 1 diabetes (Blanpain and Simons, 2013; Kimbrel and Lanza, 2015). Based on the derivation method, there are two types of PSCs, human embryonic stem cells (hESCs) and induced pluripotent stem cells (iPSCs). hESCs are derived from the inner cell mass of the normal human blastocysts or human blastocysts established with somatic cell nuclear transfer technology by inserting somatic cell nucleus into the enucleated egg (Thomson et al., 1998; Tachibana et al., 2013). iPSCs are derived by nuclear reprogramming of somatic cells with various cocktails of reprogramming factors such as OCT4, SOX2, c-MYC and KLF4 (Takahashi and Yamanaka, 2006; Yu et al., 2007; Park et al., 2008). Two studies have confirmed that mouse iPSCs are fully pluripotent as ESCs (Boland et al., 2009; Zhao et al., 2009).

Significant progress has been achieved in establishing the conditions to differentiate human PSCs (hPSCs) into various lineages of biologically active cells. For example, hESC-derived cardiomyocytes can improve cardiac function in animal models after myocardial Infarction (Passier et al., 2008). hESC-derived oligodendroglial progenitors can improve neural functions in animal models after spinal cord injury (Coutts and Keirstead, 2008). In addition, hESC-derived pancreatic β cells can restore insulin independence in Type 1 diabetes animal models (D’Amour et al., 2006; Kroon et al., 2008). Cell therapies with hESC-derived cells have entered clinical trials to treat macular degeneration, spinal cord injury and Type 1 diabetes with promising results (Angelos and Kaufman, 2015). However, the major challenge that remains for hESC-based cell therapies is the allogeneic immune rejection of hESC-derived cells by the recipients. Using humanized mouse models reconstituted with a functional human immune system, recent studies have shown that the expression of CTLA4-Ig/PD-L1 in hESC-derived cells protects these cells from allogeneic immune rejection (Rong et al., 2014). The cancer risk of these immune evasive CTLA4-Ig/PD-L1-expressing cells could be mitigated by co-expressing a suicidal gene such as thymidine kinase (He et al., 2017). Recent studies have also suggested that the cells derived from HLA-deficient hPSCs could be protected from allogeneic immune cells *in vitro* or in mouse models reconstituted with human peripheral blood cells (Xu et al., 2019), however, it remains unclear whether these cells can be protected from allogeneic immune system for an extended period of time or in humanized mice reconstituted with a more vigorous immune system.

The optimization of iPSC technology has raised the hope that the cells derived from a patient’s iPSCs can be immune tolerated by the same patient. However, published studies demonstrate that certain cells derived from iPSCs are immunogenic to the autologous immune system (Zhao et al., 2011, 2015), but certain lineages of cells derived from iPSCs could be immune tolerated by autologous immune system due to the overexpression of immune suppressive cytokines such as IL-10 (de Almeida et al., 2014). In addition, iPSCs exhibited various types of genetic instability such as somatic gene mutations and chromosome copy number variations, raising safety concerns of iPSC-based cell therapy (Mummery, 2011). In support of this notion, while autologous hiPSC-derived retinal pigmented epithelials have been tested in clinical trials to treat macular degeneration, the clinical trial was halted prematurely due to the genetic instability of hiPSCs (Mandai et al., 2017). The extensive expansion of hESCs in culture can increase genomic instability (Merkle et al., 2017). Therefore, to achieve the potential of human PSCs in cell therapy, it is critical to understand the mechanisms how PSCs maintain genomic stability.

## Tumor Suppressor p53

Since its discovery forty years ago, the tumor suppressor *p53* gene has become the most intensively studied gene with over 80,000 relevant publications. While the complex roles of p53 remain to be elucidated, p53 is known as “the guardian of the genome” and is required to maintain genomic stability of mammalian cells (Lane, 1992; Levine, 1997). Genetic instability, a hallmark of human cancer, promotes metastasis and drug-resistance (Hanahan and Weinberg, 2011). The critical roles of p53 in tumor suppression is further underscored by the findings that the *p53* gene is the most frequently mutated tumor suppressor genes in human cancers with somatic mutational rate over 50% of all human cancers (Soussi and Béroud, 2001). In addition to somatic mutation of the p53 gene, the loss of wild-type (WT) p53 functions in human cancers can also be achieved through epigenetic silencing or disruption of pathways such as the ATM pathway that are required for p53 activation after DNA damage (Inoue et al., 2012; Muller and Vousden, 2013; Jain and Barton, 2018).

Structural and functional analysis have demonstrated that p53 is a transcription factor with a sequence-specific DNA-binding domain in the central region and a transcriptional activation domain at the N-terminus (Ko and Prives, 1996). The C-terminus of p53 contains a tetramerization domain and a regulatory domain. As a transcriptional factor, p53 binds to the specific sequences in the genome and directly regulates the expression of hundreds of genes that mediate p53-dependent functions (Menendez et al., 2009). In this context, p53 activates the expression of hundred of genes, including *p21*, *MDM2*, *GADD45*, *PERP*, *NOXA* and *CYCLIN G.* In addition, p53 also suppresses the expression of some genes, such as *MAP4* and *NANOG* (Murphy et al., 1999; Lin et al., 2005). The importance of the transcriptional activity of p53 in tumor suppression is further underscored by the findings that the hotspot missense mutations of p53 in human cancers uniformly disrupt the normal DNA-binding activities of WT p53 (Weisz et al., 2007). In addition to the loss of WT p53 activity, p53 mutants also gain oncogenic activities in promoting tumorigenesis (Sabapathy and Lane, 2017).

p53 plays important roles in cellular responses to various stresses. In response to genotoxic and oncogenic stresses, p53 induces cell cycle arrest, apoptosis or senescence of the stressed somatic cells to prevent the passage of the genetic abnormalities to their offsprings, and thus maintaining the genomic stability of mammalian cells (Vousden and Prives, 2009; Zhao and Xu, 2010; Eischen, 2016). While p53-dependent apoptosis and cell cycle arrest are not required for p53-dependent tumor suppression (Janic et al., 2018), they could collaborate with DNA repair pathways to maintain genomic stability and tumor suppression (Janic et al., 2018). In addition, p53 plays complex roles in cellular metabolism, contributing to p53-dependent genomic stability and tumor suppression (Labuschagne et al., 2018; Kim et al., 2019; Li et al., 2019). In the absence of stresses, the activity of p53 is inhibited by MDM2 and MDMX, two transcriptional targets of p53, through protein-protein interaction (Hollstein et al., 1991; Kawamura et al., 2009; Marión et al., 2009; Lee et al., 2012). In addition, the protein levels of p53 are also maintained at low levels in the absence of stresses, because several E3 ligases such as MDM2 form complex with p53, leading to the ubiquitination and degradation of p53 (Brooks and Gu, 2006). Therefore, as potent negative regulators of p53 stability and activity, MDM2 and MDMX are oncogenes often overexpressed in human cancers to inhibit p53 function (Oliner et al., 2016).

**Figure 1:**
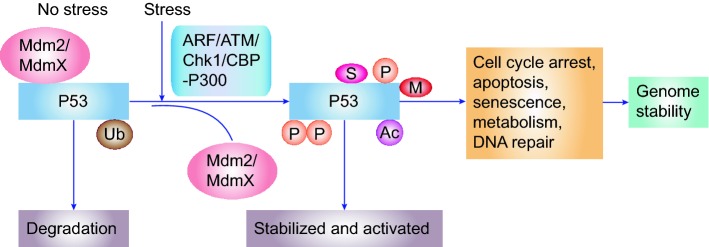
**The roles of p53 in somatic cells**. In the absence of stress, p53 is inactive and unstable due to its interaction with its transcriptional targets Mdm2/MdmX. In response to stresses, various posttranslational modifications of p53 can stabilize and activate p53 by disrupting the interaction between p53 and Mdm2/MdmX, leading to cell cycle arrest, apoptosis, senescence, DNA repair and metabolic change

Significant progress has been made to elucidate the mechanisms underlying the rapid activation of p53 in response to stresses (Fig. 1). While the mRNA levels of p53 are not significantly affected by various stresses, the rapid posttranslational modifications of p53, including phosphorylation, acetylation, methylation and sumoylation, disrupts the interaction between p53 and MDM2, leading to the stabilization and activation of p53 (Craig et al., 1999; Shieh et al., 1999; Unger et al., 1999; Wu et al., 2002; Chao et al., 2003, 2006; Song et al., 2007). In addition, the acetylation of p53 at the C-terminus promotes its DNA-binding activity and its transcriptional activity after various stresses (Gu and Roeder, 1997; Barlev et al., 2001; Feng et al., 2005; Tang et al., 2006). The posttranslational modifications of p53 play important roles in dictating the cellular responses to various stresses. For example, the phosphorylation of p53 at Ser46 primarily activates p53-dependent apoptosis after DNA damage (Saito et al., 2002; Feng et al., 2006). In addition, the phosphorylation of p53 at Ser315 is important for suppressing *NANOG* expression during the differentiation of ESCs (Lin et al., 2005). The p53 activity can also be modulated by protein-protein interaction. For example, the ASPP family proteins promote the p53-mediated apoptosis by enhancing p53-dependent induction of pro-apoptotic genes such as *PUMA* (Trigiante and Lu, 2006).

## Roles of p53 in Embryonic Stem Cells

To achieve the clinical potential of PSCs, it is required to expand PSCs for dozens of passages before their differentiation into lineage-specific functional cells. DNA damage and oncogenic pathways can be induced during the extended self-renewal and differentiation of ESCs. In this context, the rate of spontaneous mutation is significantly lower in ESCs than in somatic cells (Cervantes et al., 2002; Xu, 2005). The disruption of the p53 gene in hESCs indicates that p53 is required for maintaining the genomic stability of hESCs (Song et al., 2010). However, in contrast to somatic cells, ESCs lack p53-dependent cell cycle G_1_/S checkpoint, apoptosis, and senescence (Aladjem et al., 1998). Instead, when activated, p53 induces the differentiation of ESCs by directly suppressing the expression of the critical pluripotency factor Nanog (Lin et al., 2005). Therefore, it has been hypothesized that ESCs with unrepaired DNA damage or oncogenic stress will be eliminated from the self-renewing pool due to the reduced Nanog expression, and thus ensuring the genomic stability of self-renewing ESCs (Lin et al., 2005). Consistent with this notion, ChIP analysis of p53 and p53-dependent gene expression in ESCs indicates that p53 induces the expression of the differentiation-related genes and downregulates the pluripotency genes in response to DNA damage in ESCs (Li et al., 2012).

**Figure 2:**
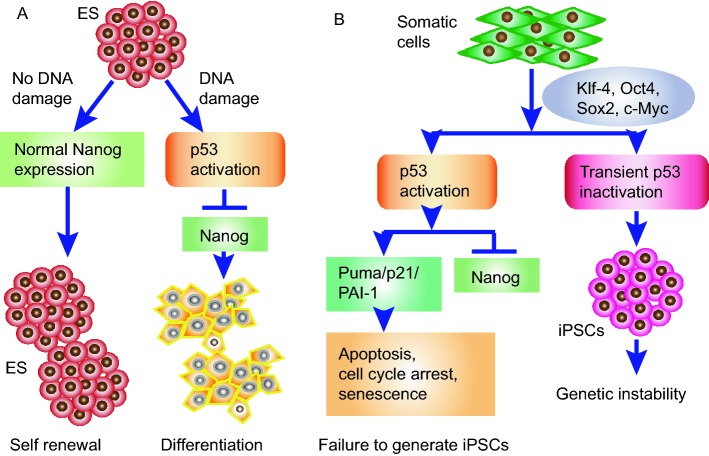
**p53 inhibits pluripotency to maintain genomic stability of pluripotent stem cells**. (A) Activation of p53 by genotoxic and oncogenic stresses in ESCs leads to the suppression of Nanog expression and the differentiation of ESCs, ensuring the genomic stability of self-renewing ESCs. (B) p53 inhibits the nuclear reprogramming of somatic cells into iPSCs. DNA damage and oncogenic stress during nuclear reprogramming activate p53, leading to cell cycle arrest, apoptosis and senescence, all of which suppress reprogramming. Transient inactivation of p53 during reprogramming will greatly improve the reprogramming efficiency at the expense of genomic stability

In response to the differentiation stimuli such as retinoic acid (RA), p53 is activated after being acetylated by CBP/p300 histone acetyl transferases to induce ESC differentiation (Jain et al., 2012). In the absence of stresses, the activity of p53 must be suppressed to maintain pluripotency. In this context, the key pluripotency factor OCT4 activates the expression of histone deacetylase SIRT1, which inactivates p53 by deacetylation of p53 (Zhang et al., 2014). The extensive culture of hESCs leads to the accumulation of hESCs harboring mutated p53, raising the cancer risk of hESCs after long-term culture (Merkle et al., 2017). In this context, certain p53 mutants have gain of functions to promote the expression of pluripotent genes and thus the preferential expansion of hESCs harboring these p53 mutants (Koifman et al., 2018). Therefore, it is important to develop culture conditions that avoid the favorable selection of hESCs harboring p53 mutations during the extended culture. In summary, p53 plays a key role in maintaining genome stability of ESCs by coordinating the DNA damage response with pluripotency (Fig. 2).

p53 also plays an important role in maintaining pluripotency by regulating the expression of genes important for pluripotency. In this context, p53 activates the expression of LIF, which is important for maintaining pluripotency of ESCs (Hu et al., 2007). p53 also regulates the expression of long non-coding RNAs (LncRNAs) that are important for pluripotency. LncRNAs are longer than 200 nucleotides and often poly-adenylated without evident ORFs (Fatica and Bozzoni, 2014; Rinn, 2014). p53 directly regulates the expression of over 40 LncRNAs in hESCs, such as HOTAIRM1 and lncPRESS (p53-regulated and ESC-associated) (Jain et al., 2016). Some LncRNAs are highly expressed in hESCs and are repressed by p53 during differentiation. For example, lncPRESS1 is involved in deacetylating H3K56ac and H3K9c in the chromatin of ESCs by releasing SIRT6 (Jain et al., 2016). In addition, lncPRESS4, also known as TUNA, is required for maintaining pluripotency by directly binding to the promoter of Nanog, Sox2 and Fgf4 (Lin et al., 2014).

Oxidative stress, a byproduct of mitochondrial oxidative phosphorylation, is the major physiological inducer of DNA damage. In this context, p53 is important to activate the expression of anti-oxidant genes and help to reduce the levels of oxidative stress (Liu and Xu, 2010). In addition, PSCs primarily rely on glycolysis for energy and substrates for biosynthesis, providing another mechanism to maintain genetic stability by minimizing the DNA damage induced by oxidative stress (Cliff and Dalton, 2017). Recent studies have demonstrated an unexpected role of p53 in suppressing oxidative phosphorylation (Kim et al., 2019). In this context, p53 activates the expression of *PUMA*, which suppresses oxidative phosphorylation by reducing the mitochondrial pyruvate uptake through the disruptive interaction between PUMA and mitochondrial pyruvate carrier complex. Therefore, p53 plays diverse roles in maintaining the genomic stability of ESCs.

## Roles of p53 in Induced Pluripotency

The breakthrough of iPSC technology was first achieved by simultaneously expressing four reprogramming factors (Oct4, Sox2, Klf-4 and c-Myc) in mouse fibroblasts (Takahashi and Yamanaka, 2006). This iPSC technology is evolutionarily conserved because the same cocktail of genes can be used to reprogram somatic cells into iPSCs of various species, including rats, monkeys and human (Trounson, 2009). Other combinations of reprogramming factors have been discovered to achieve induced pluripotency, including the cocktail of Oct4, Sox2, Lin28 and Nanog (Yu et al., 2007). Oct4 and Sox2 appear to be critical for induced pluripotency and are sufficient to reprogram progenitor cells into iPSCs (Kim et al., 2008, 2009a, b; Giorgetti et al., 2009). Small chemical cocktails can also improve the efficiency of reprogramming (Shi et al., 2008; Li et al., 2009). In theory, human iPSCs (hiPSCs) derived from patients could become a renewable source of autologous cells, and therefore, have great potential in human cell therapy. Patient-specific hiPSCs also provide a unique opportunity in modeling human diseases for mechanistic studies and drug discovery (Song et al., 2010; Boulting et al., 2011; Soldner and Jaenisch, 2012; Matsa et al., 2016).

One of the key bottlenecks for IPSC technology is the extreme low efficiency of the successful reprogramming. In search for the technology to improve the reprogramming efficiency, it has become apparent that p53 is a key bottleneck for reprogramming (Zhao et al., 2008; Banito et al., 2009; Kawamura et al., 2009; Utikal et al., 2009; Smith et al., 2010). All reprogramming factors are oncogenic and often overexpressed in human cancers, especially c-Myc and Klf4 that are potent oncoproteins (Zhao and Xu, 2010). The overexpression of such oncoproteins in somatic cells will activate p53, leading to cell cycle arrest, apoptosis and senescence that can all block successful iPSC reprogramming (Fig. 2). In addition, the activation of p53 in reprogramming cells will suppress the expression of Nanog that is required for maintaining pluripotency (Lin et al., 2005). Therefore, the silencing of the p53 gene during reprogramming has become an effective approach to increase the reprogramming efficiency (Zhao et al., 2008; Banito et al., 2009; Kawamura et al., 2009; Utikal et al., 2009; Smith et al., 2010). In addition, proteins such as Oct4 and ZSCAN4 can promote the reprogramming efficiency by inhibiting p53 (Jiang et al., 2013; Zhang et al., 2014). The silencing of the genes that are responsible for p53-dependent cell cycle arrest and apoptosis, such as *p21* and *Puma*, can also increase the frequency of nuclear reprogramming into induced pluripotency (Lake et al., 2012; Son et al., 2013).

If the transient inactivation of p53 activity is a prerequisite of successful reprogramming into iPSCs, considering the critical roles of p53 in maintaining genomic stability of mammalian cells, this raises a serious concern for the genomic instability of iPSCs. In this context, a series of studies have demonstrated that iPSCs harbor increased genetic abnormalities (Gore et al., 2011; Chen et al., 2012; Ji et al., 2012; Li et al., 2014). In addition, the identification of the oncogenic mutations harbored by iPSCs has halted the first iPSC-based clinic trial to treat macular degeneration (Mandai et al., 2017). The genomic instability could also contribute to the immunogenicity of iPSC-derived autologous cells (Robertson et al., 2007; Zhao et al., 2011; de Almeida et al., 2014; Zhao et al., 2015; Todorova et al., 2016). Recent studies also demonstrate that mitochondrial DNA mutations in iPSCs contribute to the immunogenecity of iPSCs (Deuse et al., 2019). These safety concerns must be addressed before the clinical development of iPSC-based human cell therapy.

## Concluding Remarks

Accumulating data have demonstrated that p53 is required to maintain the genomic stability of PSCs but with mechanisms distinct from somatic cells. In this context, p53 inhibits pluripotency by suppressing the expression of critical pluripotency factors, Nanog, LIF and LncRNA, and thus eliminates the stem cells with unrepaired DNA damage from the self-renewing pool. In addition, p53 activates the expression of anti-oxidant genes and suppresses oxidative phosphorylation, reducing the levels of oxidative stress that is the key physiological inducer of DNA damage. The transient loss of these roles of p53 during nuclear reprogramming of somatic cells into iPSCs contributes to genetic instability of iPSCs. The optimization of the reprogramming technology and the culture conditions of PSCs will improve the feasibility to develop PSC-based human cell therapy.

## References

[CR1] Aladjem MI, Spike BT, Rodewald LW, Hope TJ, Klemm M, Jaenisch R, Wahl GM (1998). ES cells do not activate p53-dependent stress responses and undergo p53-independent apoptosis in response to DNA damage. Curr Biol.

[CR2] Angelos MG, Kaufman DS (2015). Pluripotent stem cell applications for regenerative medicine. Curr Opin Organ Transplant.

[CR4] Banito A, Rashid ST, Acosta JC, Li S, Pereira CF, Geti I, Pinho S, Silva JC, Azuara V, Walsh M (2009). Senescence impairs successful reprogramming to pluripotent stem cells. Genes Dev.

[CR5] Barlev NA, Liu L, Chehab NH, Mansfield K, Harris KG, Halazonetis TD, Berger SL (2001). Acetylation of p53 activates transcription through recruitment of coactivators/histone acetyltransferases. Mol Cell.

[CR6] Blanpain C, Simons BD (2013). Unravelling stem cell dynamics by lineage tracing. Nat Rev Mol Cell Biol.

[CR7] Boland MJ, Hazen JL, Nazor KL, Rodriguez AR, Gifford W, Martin G, Kupriyanov S, Baldwin KK (2009). Adult mice generated from induced pluripotent stem cells. Nature.

[CR8] Boulting GL, Kiskinis E, Croft GF, Amoroso MW, Oakley DH, Wainger BJ, Williams DJ, Kahler DJ, Yamaki M, Davidow L (2011). A functionally characterized test set of human induced pluripotent stem cells. Nat Biotechnol.

[CR9] Brooks CL, Gu W (2006). p53 ubiquitination: Mdm2 and beyond. Mol Cell.

[CR10] Cervantes RB, Stringer JR, Shao C, Tischfield JA, Stambrook PJ (2002). Embryonic stem cells and somatic cells differ in mutation frequency and type. Proc Natl Acad Sci USA.

[CR11] Chao C, Hergenhahn M, Kaeser MD, Wu Z, Saito S, Iggo R, Hollstein M, Appella E, Xu Y (2003). Cell type- and promoter-specific roles of Ser18 phosphorylation in regulating p53 responses. J Biol Chem.

[CR12] Chao C, Herr D, Chun J, Xu Y (2006). Ser18 and 23 phosphorylation is required for p53-dependent apoptosis and tumor suppression. Embo J.

[CR13] Chen Z, Zhao T, Xu Y (2012). The genomic stability of induced pluripotent stem cells. Protein Cell.

[CR14] Cliff TS, Dalton S (2017). Metabolic switching and cell fate decisions: implications for pluripotency, reprogramming and development. Curr Opin Genet Dev.

[CR100] Coutts M, Keirstead HS (2008). Stem cells for the treatment of spinal cord injury. Exp Neurol.

[CR15] Craig AL, Burch L, Vojtesek B, Mikutowska J, Thompson A, Hupp TR (1999). Novel phosphorylation sites of human tumour suppressor protein p53 at Ser20 and Thr18 that disrupt the binding of mdm2 (mouse double minute 2) protein are modified in human cancers. Biochem J.

[CR101] D'Amour KA, Bang AG, Eliazer S, Kelly OG, Agulnick AD, Smart NG, Moorman MA, Kroon E, Carpenter MK, Baetge EE (2006). Production of pancreatic hormone-expressing endocrine cells from human embryonic stem cells. Nat Biotech.

[CR16] de Almeida PE, Meyer EH, Kooreman NG, Diecke S, Dey D, Sanchez-Freire V, Hu S, Ebert A, Odegaard J, Mordwinkin NM (2014). Transplanted terminally differentiated induced pluripotent stem cells are accepted by immune mechanisms similar to self-tolerance. Nat Commun.

[CR99] Deuse T, Hu X, Agbor-Enoh S, Koch M, Spitzer MH, Gravina A, Alawi M, Marishta A, Peters B, Kosaloglu-Yalcin Z (2019). De novo mutations in mitochondrial DNA of iPSCs produce immunogenic neoepitopes in mice and humans. Nat Biotechnol.

[CR17] Eischen CM (2016). Genome stability requires p53. Cold Spring Harb Perspect Med.

[CR18] Fatica A, Bozzoni I (2014). Long non-coding RNAs: new players in cell differentiation and development. Nat Rev Genet.

[CR19] Feng L, Lin T, Uranishi H, Gu W, Xu Y (2005). Functional analysis of the roles of posttranslational modifications at the p53 C terminus in regulating p53 stability and activity. Mol Cell Biol.

[CR20] Feng L, Hollstein M, Xu Y (2006). Ser46 phosphorylation regulates p53-dependent apoptosis and replicative senescence. Cell Cycle.

[CR21] Giorgetti A, Montserrat N, Aasen T, Gonzalez F, Rodríguez-Pizà I, Vassena R, Raya A, Boué S, Barrero MJ, Corbella BA (2009). Generation of induced pluripotent stem cells from human cord blood using OCT4 and SOX2. Cell Stem Cell.

[CR22] Gore A, Li Z, Fung H-L, Young JE, Agarwal S, Antosiewicz-Bourget J, Canto I, Giorgetti A, Israel MA, Kiskinis E (2011). Somatic coding mutations in human induced pluripotent stem cells. Nature.

[CR23] Gu W, Roeder RG (1997). Activation of p53 sequence-specific DNA binding by acetylation of the p53 C-terminal domain. Cell.

[CR24] Hanahan D, Weinberg RA (2011). Hallmarks of cancer: the next generation. Cell.

[CR25] He J, Rong Z, Fu X, Xu Y (2017). A safety checkpoint to eliminate cancer risk of the immune evasive cells derived from human embryonic stem cells. Stem Cells.

[CR26] Hollstein M, Sidransky D, Vogelstein B, Harris CC (1991). p53 mutations in human cancers. Science.

[CR27] Hu W, Feng Z, Teresky AK, Levine AJ (2007). p53 regulates maternal reproduction through LIF. Nature.

[CR28] Inoue K, Kurabayashi A, Shuin T, Ohtsuki Y, Furihata M (2012). Overexpression of p53 protein in human tumors. Med Mol Morphol.

[CR29] Jain AK, Barton MC (2018). P53: emerging roles in stem cells, development and beyond. Development.

[CR30] Jain AK, Allton K, Iacovino M, Mahen E, Milczarek RJ, Zwaka TP, Kyba M, Barton MC (2012). p53 regulates cell cycle and microRNAs to promote differentiation of human embryonic stem cells. PLoS Biol.

[CR31] Jain AK, Xi Y, McCarthy R, Allton K, Akdemir KC, Patel LR, Aronow B, Lin C, Li W, Yang L (2016). LncPRESS1 Is a p53-regulated LncRNA that safeguards pluripotency by disrupting SIRT6-mediated de-acetylation of histone H3K56. Mol Cell.

[CR32] Janic A, Valente LJ, Wakefield MJ, Di Stefano L, Milla L, Wilcox S, Yang H, Tai L, Vandenberg CJ, Kueh AJ (2018). DNA repair processes are critical mediators of p53-dependent tumor suppression. Nat Med.

[CR33] Ji J, Ng SH, Sharma V, Neculai D, Hussein S, Sam M, Trinh Q, Church GM, McPherson JD, Nagy A (2012). Elevated coding mutation rate during the reprogramming of human somatic cells into induced pluripotent stem cells. Stem Cells.

[CR34] Jiang J, Lv W, Ye X, Wang L, Zhang M, Yang H, Okuka M, Zhou C, Zhang X, Liu L (2013). Zscan4 promotes genomic stability during reprogramming and dramatically improves the quality of iPS cells as demonstrated by tetraploid complementation. Cell Res.

[CR35] Kawamura T, Suzuki J, Wang YV, Menendez S, Morera LB, Raya A, Wahl GM, Belmonte JCI (2009). Linking the p53 tumour suppressor pathway to somatic cell reprogramming. Nature.

[CR36] Kim JB, Zaehres H, Wu G, Gentile L, Ko K, Sebastiano V, Araúzo-Bravo MJ, Ruau D, Han DW, Zenke M (2008). Pluripotent stem cells induced from adult neural stem cells by reprogramming with two factors. Nature.

[CR37] Kim JB, Sebastiano V, Wu G, Araúzo-Bravo MJ, Sasse P, Gentile L, Ko K, Ruau D, Ehrich M, van den Boom D (2009). Oct4-induced pluripotency in adult neural stem cells. Cell.

[CR38] Kim JV, Kang SS, Dustin ML, McGavern DB (2009). Myelomonocytic cell recruitment causes fatal CNS vascular injury during acute viral meningitis. Nature.

[CR39] Kim J, Yu L, Chen W, Xu Y, Wu M, Todorova D, Tang Q, Feng B, Jiang L, He J (2019). Wild-type p53 promotes cancer metabolic switch by inducing PUMA-dependent suppression of oxidative phosphorylation. Cancer Cell.

[CR40] Kimbrel EA, Lanza R (2015). Current status of pluripotent stem cells: moving the first therapies to the clinic. Nat Rev Drug Discov.

[CR41] Ko LJ, Prives C (1996). p53: puzzle and paradigm. Genes Dev.

[CR42] Koifman G, Shetzer Y, Eizenberger S, Solomon H, Rotkopf R, Molchadsky A, Lonetto G, Goldfinger N, Rotter V (2018). A mutant p53-dependent embryonic stem cell gene signature is associated with augmented tumorigenesis of stem cells. Cancer Res.

[CR102] Kroon E, Martinson LA, Kadoya K, Bang AG, Kelly OG, Eliazer S, Young H, Richardson M, Smart NG, Cunningham J (2008). Pancreatic endoderm derived from human embryonic stem cells generates glucose-responsive insulin-secreting cells in vivo. Nat Biotech.

[CR43] Labuschagne CF, Zani F, Vousden KH (2018). Control of metabolism by p53: cancer and beyond. Biochim Biophys Acta.

[CR44] Lake BB, Fink J, Klemetsaune L, Fu X, Jeffers JR, Zambetti GP, Xu Y (2012). Context-dependent enhancement of induced pluripotent stem cell reprogramming by silencing Puma. Stem cells.

[CR45] Lane DP (1992). p53, guardian of the genome. Nature.

[CR46] Lee D-F, Su J, Ang Y-S, Carvajal-Vergara X, Mulero-Navarro S, Pereira Carlos F, Gingold J, Wang H-L, Zhao R, Sevilla A (2012). Regulation of embryonic and induced pluripotency by aurora kinase-p53 signaling. Cell Stem Cell.

[CR47] Levine AJ (1997). p53, the cellular gatekeeper for growth and division. Cell.

[CR48] Li W, Wei W, Zhu S, Zhu J, Shi Y, Lin T, Hao E, Hayek A, Deng H, Ding S (2009). Generation of rat and human induced pluripotent stem cells by combining genetic reprogramming and chemical inhibitors. Cell Stem Cell.

[CR49] Li M, He Y, Dubois W, Wu X, Shi J, Huang J (2012). Distinct regulatory mechanisms and functions for p53-activated and p53-repressed DNA damage response genes in embryonic stem cells. Mol Cell.

[CR50] Li Z, Lu H, Yang W, Yong J, Zhang Z-N, Zhang K, Deng H, Xu Y (2014). Mouse SCNT ESCs have lower somatic mutation load than syngeneic iPSCs. Stem Cell Rep.

[CR51] Li L, Mao Y, Zhao L, Li L, Wu J, Zhao M, Du W, Yu L, Jiang P (2019). p53 regulation of ammonia metabolism through urea cycle controls polyamine biosynthesis. Nature.

[CR52] Lin T, Chao C, Saito SI, Mazur SJ, Murphy ME, Appella E, Xu Y (2005). p53 induces differentiation of mouse embryonic stem cells by suppressing Nanog expression. Nat Cell Biol.

[CR53] Lin N, Chang K-Y, Li Z, Gates K, Rana Zacharia A, Dang J, Zhang D, Han T, Yang C-S, Cunningham Thomas J (2014). An evolutionarily conserved long noncoding RNA TUNA controls pluripotency and neural lineage commitment. Mol Cell.

[CR54] Liu D, Xu Y (2010). p53, oxidative stress, and aging. Antioxid Redox Signal.

[CR55] Mandai M, Watanabe A, Kurimoto Y, Hirami Y, Morinaga C, Daimon T, Fujihara M, Akimaru H, Sakai N, Shibata Y (2017). Autologous induced stem-cell-derived retinal cells for macular degeneration. N Engl J Med.

[CR56] Marión RM, Strati K, Li H, Murga M, Blanco R, Ortega S, Fernandez-Capetillo O, Serrano M, Blasco MA (2009). A p53-mediated DNA damage response limits reprogramming to ensure iPS cell genomic integrity. Nature.

[CR57] Matsa E, Ahrens JH, Wu JC (2016). Human induced pluripotent stem cells as a platform for personalized and precision cardiovascular medicine. Physiol Rev.

[CR58] Menendez D, Inga A, Resnick MA (2009). The expanding universe of p53 targets. Nat Rev Cancer.

[CR59] Merkle FT, Ghosh S, Kamitaki N, Mitchell J, Avior Y, Mello C, Kashin S, Mekhoubad S, Ilic D, Charlton M (2017). Human pluripotent stem cells recurrently acquire and expand dominant negative P53 mutations. Nature.

[CR60] Muller PAJ, Vousden KH (2013). p53 mutations in cancer. Nature Cell Biology.

[CR61] Mummery C (2011). Induced pluripotent stem cells—a cautionary note. N Engl J Med.

[CR62] Murphy M, Ahn J, Walker KK, Hoffman WH, Evans RM, Levine AJ, George DL (1999). Transcriptional repression by wild-type p53 utilizes histone deacetylases, mediated by interaction with mSin3a. Genes Dev.

[CR63] Oliner JD, Saiki AY, Caenepeel S (2016). The role of MDM2 amplification and overexpression in tumorigenesis. Cold Spring Harb Perspect Med.

[CR64] Park IH, Zhao R, West JA, Yabuuchi A, Huo H, Ince TA, Lerou PH, Lensch MW, Daley GQ (2008). Reprogramming of human somatic cells to pluripotency with defined factors. Nature.

[CR103] Passier R, van Laake LW, Mummery CL (2008). Stem-cell-based therapy and lessons from the heart. Nature.

[CR65] Rinn JL (2014). lncRNAs: linking RNA to chromatin. Cold Spring Harb Perspect Biol.

[CR66] Robertson NJ, Brook FA, Gardner RL, Cobbold SP, Waldmann H, Fairchild PJ (2007). Embryonic stem cell-derived tissues are immunogenic but their inherent immune privilege promotes the induction of tolerance. Proc Natl Acad Sci USA.

[CR67] Rong Z, Wang M, Hu Z, Stradner M, Zhu S, Kong H, Yi H, Goldrath A, Yang Y-G, Xu Y (2014). An effective approach to prevent immune rejection of human ESC-derived allografts. Cell Stem Cell.

[CR68] Sabapathy K, Lane DP (2017). Therapeutic targeting of p53: all mutants are equal, but some mutants are more equal than others. Nat Rev Clin Oncol.

[CR69] Saito S, Goodarzi AA, Hagashimoto Y, Noda Y, Lees-Miller SP, Appella E, Anderson CW (2002). ATM mediates phosphorylation at multiple p53 sites, including Ser46, in response to ionizing radiation. J Biol Chem.

[CR70] Shi Y, Desponts C, Do JT, Hahm HS, Schˆler HR, Ding S (2008). Induction of pluripotent stem cells from mouse embryonic fibroblasts by Oct4 and Klf4 with small-molecule compounds. Cell Stem Cell.

[CR71] Shieh SY, Taya Y, Prives C (1999). DNA damage-inducible phosphorylation of p53 at N-terminal sites including a novel site, Ser20, requires tetramerization. Embo J.

[CR72] Smith ZD, Nachman I, Regev A, Meissner A (2010). Dynamic single-cell imaging of direct reprogramming reveals an early specifying event. Nat Biotechnol.

[CR73] Soldner F, Jaenisch R (2012). iPSC disease modeling. Science.

[CR74] Son MJ, Son MY, Seol B, Kim MJ, Yoo CH, Han MK, Cho YS (2013). Nicotinamide overcomes pluripotency deficits and reprogramming barriers. Stem Cells.

[CR75] Song J, Chao C, Xu Y (2007). Ser18 and Ser23 phosphorylation plays synergistic roles in activating p53-dependent neuronal apoptosis. Cell Cycle.

[CR76] Song H, Chung S-K, Xu Y (2010). Modeling disease in human ESCs using an efficient BAC-based homologous recombination system. Cell Stem Cell.

[CR77] Soussi T, Béroud C (2001). Assessing TP53 status in human tumours to evaluate clinical outcome. Nat Rev Cancer.

[CR78] Tachibana M, Amato P, Sparman M, Gutierrez Nuria M, Tippner-Hedges R, Ma H, Kang E, Fulati A, Lee H-S, Sritanaudomchai H (2013). Human embryonic stem cells derived by somatic cell nuclear transfer. Cell.

[CR79] Takahashi K, Yamanaka S (2006). Induction of pluripotent stem cells from mouse embryonic and adult fibroblast cultures by defined factors. Cell.

[CR80] Tang Y, Luo J, Zhang W, Gu W (2006). Tip60-dependent acetylation of p53 modulates the decision between cell-cycle arrest and apoptosis. Mol Cell.

[CR81] Thomson JA, Itskovitz-Eldor J, Shapiro SS, Waknitz MA, Swiergiel JJ, Marshall VS, Jones JM (1998). Embryonic stem cell lines derived from human blastocysts. Science.

[CR82] Todorova D, Kim J, Hamzeinejad S, He J, Xu Y (2016). Brief report: immune microenvironment determines the immunogenicity of induced pluripotent stem cell derivatives. Stem Cells.

[CR83] Trigiante G, Lu X (2006). ASPP [corrected] and cancer. Nat Rev Cancer.

[CR84] Trounson A (2009). Rats, cats, and elephants, but still no unicorn: induced pluripotent stem cells from new species. Cell Stem Cell.

[CR85] Unger T, Juven-Gershon T, Moallem E, Berger M, Vogt Sionov R, Lozano G, Oren M, Haupt Y (1999). Critical role for Ser20 of human p53 in the negative regulation of p53 by Mdm2. Embo J.

[CR86] Utikal J, Polo JM, Stadtfeld M, Maherali N, Kulalert W, Walsh RM, Khalil A, Rheinwald JG, Hochedlinger K (2009). Immortalization eliminates a roadblock during cellular reprogramming into iPS cells. Nature.

[CR87] Vousden KH, Prives C (2009). Blinded by the light: the growing complexity of p53. Cell.

[CR88] Weisz L, Oren M, Rotter V (2007). Transcription regulation by mutant p53. Oncogene.

[CR89] Wu Z, Earle J, Saito S, Anderson CW, Appella E, Xu Y (2002). Mutation of mouse p53 Ser23 and the response to DNA damage. Mol Cell Biol.

[CR90] Xu Y (2005). A new role of p53 in maintaining genetic stability in embryonic stem cells. Cell Cycle.

[CR91] Xu H, Wang B, Ono M, Kagita A, Fujii K, Sasakawa N, Ueda T, Gee P, Nishikawa M, Nomura M (2019). Targeted disruption of HLA genes via CRISPR-Cas9 generates iPSCs with enhanced immune compatibility. Cell Stem Cell.

[CR92] Yu J, Vodyanik MA, Smuga-Otto K, Antosiewicz-Bourget J, Frane JL, Tian S, Nie J, Jonsdottir GA, Ruotti V, Stewart R (2007). Induced pluripotent stem cell lines derived from human somatic cells. Science.

[CR93] Zhang Z-N, Chung S-K, Xu Z, Xu Y (2014). Oct4 maintains the pluripotency of human embryonic stem cells by inactivating p53 through Sirt1-mediated deacetylation. Stem Cells.

[CR94] Zhao T, Xu Y (2010). P53 and stem cells: new developments and new concerns. Trends Cell Biol.

[CR95] Zhao Y, Yin X, Qin H, Zhu F, Liu H, Yang W, Zhang Q, Xiang C, Hou P, Song Z (2008). Two supporting factors greatly improve the efficiency of human iPSC generation. Cell Stem Cell.

[CR96] Zhao XY, Li W, Lv Z, Liu L, Tong M, Hai T, Hao J, Guo CL, Ma QW, Wang L (2009). iPS cells produce viable mice through tetraploid complementation. Nature.

[CR97] Zhao T, Zhang Z-N, Rong Z, Xu Y (2011). Immunogenicity of induced pluripotent stem cells. Nature.

[CR98] Zhao T, Zhang Z-N, Westenskow PD, Todorova D, Hu Z, Lin T, Rong Z, Kim J, He J, Wang M (2015). Humanized mice reveal differential immunogenicity of cells derived from autologous induced pluripotent stem cells. Cell Stem Cell.

